# Causal effects of breastfeeding on childhood health outcomes: A Mendelian randomization analysis of mental and physical health

**DOI:** 10.1097/MD.0000000000044888

**Published:** 2025-11-07

**Authors:** Yue Lu, Xi Yu, Zijuan Feng, Zheng Xiang, Fan Xu, Jiang Zheng, Shunli Rui, Xiaomei Yue, Yao Zhao

**Affiliations:** a Department of Pediatric Research Institute Children’s Hospital of Chongqing Medical University, National Clinical Research Center for Child Health and Disorders, Ministry of Education Key Laboratory of Child Development and Disorders, Chongqing Key Laboratory of Child Rare Diseases in Infection and Immunity, Chongqing, China; b Chongqing Key Laboratory of Emergency Medicine, Chongqing Emergency Medical Center, School of Medicine, Chongqing University Central Hospital, Chongqing University, Chongqing, China; c Department of Anesthesiology, Chongqing Emergency Medical Center, Chongqing University Central Hospital, School of Medicine, Chongqing University, Chongqing, China; d Department of Endocrinology and Metabolism, School of Medicine, Chongqing University Central Hospital, Chongqing Emergency Medical Centre, Chongqing University, Chongqing, China; e Department of Pathology and Clinical Laboratory, 63650 Military Hospital, Xinjiang, China.

**Keywords:** breastfeeding, Mendelian randomization, physical and mental health

## Abstract

Breastfeeding has been extensively documented to confer numerous health benefits. However, establishing causal relationships, particularly with respect to childhood mental and physical health, has been challenging due to confounding variables inherent in observational studies. This study leverages a Mendelian randomization (MR) approach to rigorously investigate the causal impacts of breastfeeding on various childhood health outcomes. A total of 49 single-nucleotide polymorphisms were chosen as instrumental variables after employing linkage disequilibrium screening to ensure strong associations with breastfeeding and minimal confounding. Genetic associations and outcome data were extracted from publicly available genome-wide association studies. Primary MR analyses were conducted using the inverse-variance weighted (IVW) method, supplemented by MR-Egger, weighted median, weighted mode, and simple mode analyses to assess sensitivity. The MR analysis did not reveal significant causal relationships between breastfeeding and childhood mental health outcomes, which included intelligence (*P*_IVW_ = .165), absence epilepsy (*P*_IVW_ = .894), social disorders (*P*_IVW_ = .781), and emotional disorders (*P*_IVW_ = .598). Nevertheless, a significant protective effect of breastfeeding on the risk of childhood asthma was observed (*P*_IVW_ = .033, odds ratio: 0.997, 95% confidence interval: 0.936–0.997). No causal associations were identified for other physical health outcomes, such as height, sunburn, obesity, and allergy. Sensitivity analyses corroborated these findings, indicating no pleiotropic effects. This MR study provides robust evidence supporting a protective effect of breastfeeding against childhood asthma. However, breastfeeding does not appear to significantly influence other mental or physical health outcomes. These findings highlight the critical public health implications of breastfeeding for reducing the incidence of childhood asthma. Further investigations are warranted across diverse populations to generalize these results and elucidate underlying biological mechanisms.

## 1. Introduction

Breastfeeding is universally recognized as the optimal form of nutrition for infants, with substantial empirical evidence underscoring its myriad health benefits for both mothers and infants.^[1]^ The World Health Organization and the American Academy of Pediatrics advocate for exclusive breastfeeding during the first 6 months of life, followed by continued breastfeeding in conjunction with appropriate complementary foods for up to 2 years or beyond.^[2,3]^ These recommendations are predicated on the extensively documented short- and long-term health outcomes associated with breastfeeding, which encompass reduced infant morbidity and mortality rates, as well as the promotion of healthy growth and development.^[4–6]^ Despite the compelling evidence favoring breastfeeding, global initiation and maintenance rates are suboptimal. The barriers to optimal breastfeeding practices include cultural norms, insufficient support and education, and socioeconomic factors.^[7,8]^ Therefore, elucidating the specific causal relationships between breastfeeding and various health outcomes is critical for reinforcing public health messaging and devising effective interventions to bolster breastfeeding practices.^[9,10]^

Most research on breastfeeding is observational in nature, which intrinsically involves confounding variables and potential reverse causality. For instance, factors such as maternal education, socioeconomic status, and access to healthcare can impact both breastfeeding practices and infant health outcomes, complicating the interpretation of findings.^[11,12]^ Although a plethora of observational studies have reported beneficial associations between breastfeeding and various cognitive and physical health outcomes, disentangling these relationships to establish causality poses significant methodological challenges.^[13,14]^

Mendelian randomization (MR) employs genetic variants associated with an exposure (in this case, breastfeeding) as instrumental variables (IVs) to infer causal effects.^[15,16]^ This approach is analogous to randomized controlled trials, given that the random allocation of alleles during meiosis mitigates confounding and reverse causality, thereby facilitating more robust causal inferences. Recent advancements in genomics and the availability of large-scale genome-wide association studies (GWAS) have propelled the application of MR in various domains, including nutrition and public health.^[17,18]^ In the context of breastfeeding, MR offers a promising avenue for elucidating causal impacts on both mental and physical health outcomes in children. Prior MR studies have shed light on the genetic determinants of breastfeeding behavior and its potential influence on child health. However, comprehensive analyses encompassing a broad array of health outcomes remain limited.^[19–21]^ By utilizing MR, researchers can overcome the limitations of observational studies and generate more reliable evidence to inform public health interventions.^[22,23]^

This study aims to examine the causal effects of breastfeeding on mental and physical health outcomes in childhood using MR. Specifically, we investigate the impact of breastfeeding on intelligence, absence epilepsy, social disorders, emotional disorders, height, sunburn, obesity, allergy, and asthma. These outcomes were selected based on prior observational evidence suggesting potential associations with breastfeeding, thus warranting further exploration through causal inference methods.^[2,4,24]^ By leveraging genetic variants as IVs, we aim to provide robust evidence that addresses the limitations of observational research, thereby contributing to a more accurate understanding of the health benefits associated with breastfeeding. The findings from this study have the potential to reinforce public health recommendations and support breastfeeding promotion programs, ultimately improving health outcomes for children on a global scale.^[5,25–27]^ We anticipate that the results will highlight specific health domains where breastfeeding exerts a significant causal effect, informing targeted interventions and strategies to enhance breastfeeding practices.^[28,29]^

## 2. Methods

### 2.1. Study design

**Figure 1.**
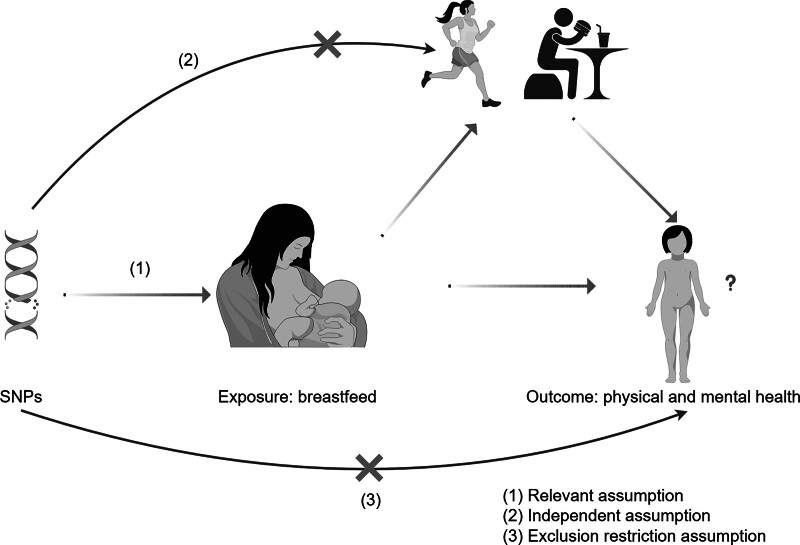
Flow chart: (A) Relevant assumption: IVs are closely related to exposure factors. (B) Independent assumption: IVs are not related to confounding factors that may affect exposure factors and outcomes. (C) Exclusion restriction assumption: IVs only affect outcomes through exposure factors (created by Figdraw). IVs = instrumental variables.

This study adheres to the 3 fundamental assumptions of MR studies: the IVs are closely associated with breastfeeding (or the physical and mental health of children); the IVs are not associated with confounders that might affect either the physical and mental health of children or breastfeeding; and the IVs influence the physical and mental health of children exclusively through the pathway of breastfeeding and not through other means (see Fig. 1). A 2-sample MR approach was utilized, leveraging summary genetic associations from GWAS related to breastfeeding, mental health factors (intelligence, absence epilepsy, social disorders, and emotional disorders), and physical health factors (height, sunburn, obesity, allergy, and asthma).^[30]^

### 2.2. Exposures

Breastfeeding status was determined by the response to the question, “Were you breastfed when you were a baby?” with response options including “Yes,” “No,” “Do not know,” and “Prefer not to answer.” Specific measures of breastfeeding duration and intensity were not available. It is important to note that breastfeeding status was determined based on self-reported data, without further specification on duration or exclusivity. As a result, this may limit the ability to detect more nuanced or dose-dependent effects of breastfeeding on childhood health outcomes.

### 2.3. Data sources

Genetic associations between exposures and outcomes were assessed using publicly accessible GWAS data. Independent instrumental single-nucleotide polymorphisms (SNPs) associated with early-life breastfeeding and various mental and physical health indicators (intelligence, absence epilepsy, social disorders, emotional disorders, height, sunburn, obesity, allergy, and asthma) in individuals of European descent were extracted from GWAS summary datasets available through the UK Biobank.^[31]^ The UK Biobank provides an extensive repository of genetic data and health information from approximately 500,000 participants from the UK and Finland, supporting significant contributions to research and advancements in human health. Table S1, Supplemental Digital Content, https://links.lww.com/MD/Q268 provides additional details regarding the data resources used.

### 2.4. IV selection

The instrumental SNPs for the exposure of interest were identified through a meticulous process adhering to 3 key assumptions. First, independent SNPs from the GWAS for breastfeeding were selected based on stringent criteria, including a *P*-value threshold of <5e‐8, linkage disequilibrium *r*^2^ < 0.001, and a window size of 10,000 kb to ensure robust associations with the exposure while minimizing linkage disequilibrium effects. Second, SNPs associated with potential confounding traits were excluded using phenome-wide scanning from the Phenome Scanner database.^[32]^ Lastly, SNPs with significant associations with the outcomes of interest (*P* < 5e‐8) were excluded from the analysis.

### 2.5. MR analysis

Primary analyses employed the inverse-variance weighted (IVW) method, utilizing a multiplicative random-effects IVW model in instances of heterogeneity, transitioning to a fixed-effect IVW model otherwise.^[33]^ Additional methods, including MR-Egger, simple mode, weighted median, and weighted mode, were employed to enhance result validity. Sensitivity analyses, such as the Cochran *Q* test and MR-Egger intercept, were separately conducted to detect any potential heterogeneity and directional pleiotropy within the IVW model. A significance level of *P* < .05 was considered indicative of potential heterogeneity or horizontal pleiotropy. The MR-Egger method assesses pleiotropy through the intercept term; an intercept term of zero suggests congruence with IVW findings, indicating the absence of horizontal pleiotropy. Additionally, a leave-one-out approach was employed to determine if any single SNP disproportionately influenced the aggregate IVW estimate.^[34]^

A statistically significant result was indicated by a *P*-value < .05. Analyses were executed using the SampleMR package (v0.5.7) in R software (v4.3.1, Boston), with data visualization performed using GraphPad Prism for Windows (Version 9.0, San Diego).

### 2.6. Ethical approval

All analyses conducted in this study utilized publicly available GWAS summary statistics. As the data had already received approval from the relevant ethics committee, no further ethical approval was necessary.

## 3. Results

### 3.1. IV selection and sensitivity analysis results

In this study, a total of 49 single SNPs meeting the significance threshold were selected as IVs following linkage disequilibrium screening (Table S2, Supplemental Digital Content, https://links.lww.com/MD/Q268). Analysis using PhenoScanner indicated that none of these 49 SNPs exhibited strong associations with the outcomes, and no confounding variables were identified. Additionally, all SNPs had *F*-values exceeding 10, resulting in the retention of all 49 SNPs. These IVs were subsequently extracted from the respective outcome databases, with the exclusion of palindromic SNPs possessing moderate allele frequencies. The final selection of SNPs for MR analysis is detailed in Table S3, Supplemental Digital Content, https://links.lww.com/MD/Q268.

### 3.2. Effect of breastfeeding on childhood mental health

**Figure 2.**
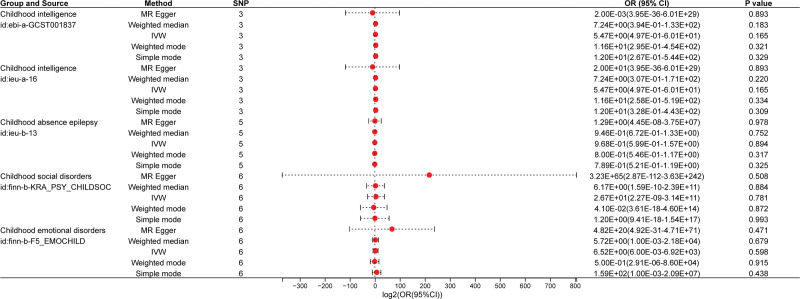
Forest plots of MR estimates for the associations between breastfeed and childhood mental health using 5 MR methods. OR = odds ratio, CI = confidence interval, SNP = single-nucleotide polymorphism.

The IVW method was utilized as the primary analytical approach for evaluating the relationship between breastfeeding and childhood mental health outcomes, including intelligence, absence epilepsy, social disorders, and emotional disorders. The findings indicated that breastfeeding did not have a statistically significant impact on intelligence (*P*_IVW_ = .165), absence epilepsy (*P*_IVW_ = .894), social disorders (*P*_IVW_ = .781), or emotional disorders (*P*_IVW_ = .598). Consistent results were obtained through MR-Egger regression, weighted median, weighted mode, and simple mode analyses (Fig. 2).

### 3.3. Effect of breastfeeding on childhood physical health

**Figure 3.**
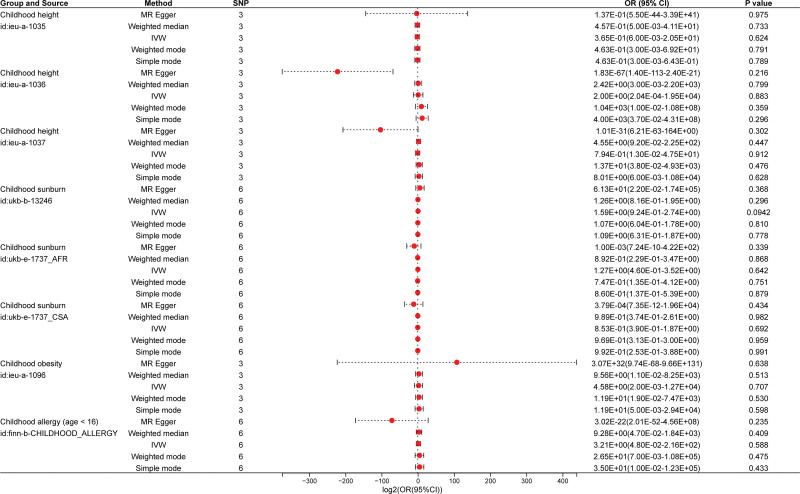
Forest plots of MR estimates for the associations between breastfeed and childhood physical health using 5 MR methods. OR = odds ratio, CI = confidence interval, SNP = single-nucleotide polymorphism.

Subsequently, MR analysis was conducted to investigate the impact of breastfeeding on various childhood physical health outcomes, including height, sunburn, obesity, allergy, and asthma. The findings revealed a statistically significant reduction in the risk of childhood asthma associated with breastfeeding (*P*_IVW_ = .033), whereas no significant causal relationships were observed for height (*P*_IVW_ = .624, *P*_IVW_ = .883, *P*_IVW_ = .912), sunburn (*P*_IVW_ = .942, *P*_IVW_ = .642, *P*_IVW_ = .692), obesity (*P*_IVW_ = .707), or allergy (*P*_IVW_ = .588). These results are graphically depicted in forest plots (Fig. 3).

**Figure 4.**
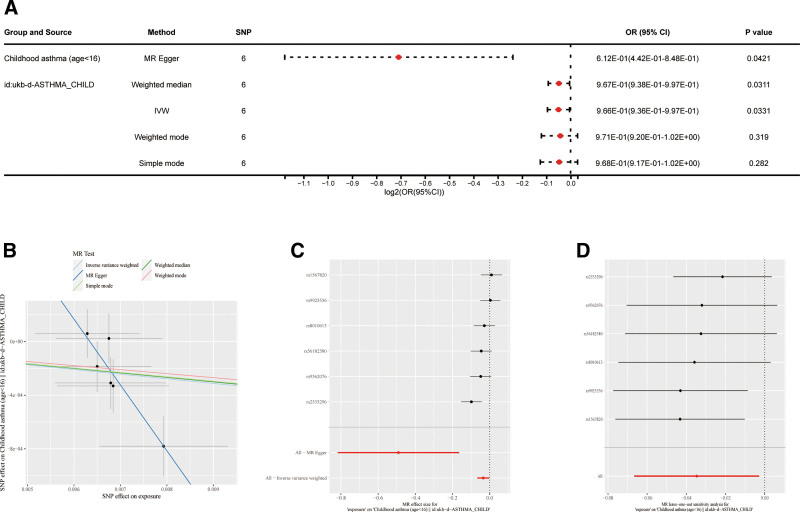
MR results of the breastfeed-childhood asthma study. (A) Forest plot of the Mendelian randomization results using 5 MR methods. (B) Scatter plot of Mendelian randomization results. (C) The causal effect of exposure on outcome is estimated using each SNP singly using the Wald ratio and represented in a forest plot. The MR estimate using all SNPs using the MR methods are also shown. (D) Leave-one-out sensitivity analysis is performed to ascertain if an association is being disproportionately influenced by a single SNP. Each black point in the forest plot represents the MR analysis excluding that particular SNP. SNP = single-nucleotide polymorphism.

The analysis showed a notable link between genetically predicted breastfeeding and a reduced risk of childhood asthma (*P*_IVW_ = .0331, odds ratio: 0.997, 95% confidence interval: 0.936–0.997). Heterogeneity was detected through Cochran *Q* test (MR-Egger: *Q* = 2.213, *P* = .697; IVW: *Q* = 9.754, *P* = .0825) (Table S4, Supplemental Digital Content, https://links.lww.com/MD/Q268). Importantly, the absence of horizontal pleiotropy as indicated by the MR-Egger intercept test (*P* = .0516) suggests that the observed associations were not influenced by pleiotropic effects (Table S5, Supplemental Digital Content, https://links.lww.com/MD/Q268). The leave-one-out sensitivity analysis indicated that no individual SNP significantly influenced the causal effect. The impacts of each SNP on the outcome were evaluated and visualized in forest plots (Fig. 4).

## 4. Discussion

In this comprehensive MR study, we sought to delineate the causal effects of breastfeeding on both mental and physical health outcomes in offspring. Our findings demonstrate no significant causal associations between breastfeeding and childhood mental health outcomes, including intelligence, absence epilepsy, social disorders, and emotional disorders. Conversely, a significant protective effect of breastfeeding against the risk of childhood asthma was observed. These findings are consistent with, yet expand upon, prior investigations aimed at elucidating these complex relationships.

Our MR analysis regarding childhood mental health revealed a lack of significant causal associations, which aligns with the inconsistencies observed in previous observational studies. For example, meta-analyses and systematic reviews have demonstrated varied outcomes regarding the association between breastfeeding and cognitive development, with some studies reporting modest benefits and others finding no substantial relationship.^[4,35,36]^ The absence of a significant effect in our study might be attributable to the rigorous control for confounding inherent in the MR approach, suggesting that observed associations in observational studies may be confounded by socio-economic and environmental factors.^[2,37]^ Similarly, the null results for absence epilepsy, social disorders, and emotional disorders underscore the importance of cautious interpretation of observational data and highlight the strength of MR studies in establishing more robust causal inferences.^[38]^

Our study’s finding of a strong link between breastfeeding and reduced childhood asthma risk is important and calls for further investigation into the biological mechanisms involved. Breast milk is known to contain immunoglobulins, anti-inflammatory agents, and bioactive compounds that may help lower the risk of asthma by influencing the infant’s immune system.^[39–42]^ These bioactive compounds are key in shaping the infant’s gut microbiota, which plays an essential role in immune system development and function.^[43–46]^ The mechanistic insights provided by our findings are consistent with studies demonstrating correlations between breastfeeding and a lower incidence of respiratory infections and other immune-related disorders in early childhood.^[47–49]^ Our findings carry significant implications for public health policies and breastfeeding promotion programs. Despite extensive evidence supporting the health benefits of breastfeeding, global breastfeeding rates remain suboptimal.^[50–52]^ This study reinforces the critical role of breastfeeding in potentially mitigating the risk of childhood asthma, thereby underscoring the necessity for enhanced efforts to support and encourage breastfeeding practices. Healthcare providers and policymakers should integrate these findings when designing and implementing interventions aimed at increasing breastfeeding initiation and duration among new mothers.^[5,53,54]^

While our study offers robust insights and enriches the evidence base regarding the health impacts of breastfeeding, it is not devoid of limitations. One key limitation is the reliance on self-reported breastfeeding status, which could lead to recall bias. While genetic instrumental variables in the MR analysis help reduce this bias, the lack of detailed data on breastfeeding duration and exclusivity may limit detection of dose-dependent effects and affect result precision. Another constraint is that the focus on individuals of European ancestry may limit the applicability of our findings to other ethnic groups. Future research should include diverse populations to verify if these results are consistent across various genetic backgrounds. While MR is effective for inferring causality, it can be affected by biases like pleiotropy, where genetic variants impact multiple traits. Our sensitivity analyses, including MR-Egger and leave-one-out methods, found no significant pleiotropy, but we cannot rule it out completely.^[22]^ We acknowledge the risk of false positives due to multiple outcomes and used a conservative significance threshold (*P* < .05), considering the broader evidence. Future research should employ stricter corrections like Bonferroni or false discovery rate control for more robust results. Additionally, larger sample sizes and refined genetic instruments are recommended for further validation.

## 5. Conclusion

In conclusion, this extensive MR analysis provides compelling evidence for a protective effect of breastfeeding against childhood asthma while indicating no significant effect on childhood mental health outcomes. These findings underscore the pivotal importance of breastfeeding as a public health intervention and emphasize the need for ongoing research to unravel the intricate biological mechanisms underlying these associations. Expanding the scope of future studies to include diverse populations and advanced genetic methodologies will be critical in elucidating the multifaceted benefits of breastfeeding and informing global health policies.

## Acknowledgments

We would like to sincerely appreciate all GWAS participants and researchers for publicly sharing the summary statistics.

## Author contributions

**Conceptualization:** Yue Lu, Yao Zhao.

**Data curation:** Yue Lu, Xi Yu.

**Formal analysis:** Zijuan Feng.

**Investigation:** Zheng Xiang.

**Methodology:** Fan Xu, Jiang Zheng, Xiaomei Yue.

**Software:** Shunli Rui.

**Supervision:** Yao Zhao.

**Validation:** Yue Lu.

**Visualization:** Yue Lu.

**Writing – original draft:** Yue Lu.

**Writing – review & editing:** Yao Zhao.

## Supplementary Material


