# Supplementary material for: Causal effects of breastfeeding on childhood health outcomes: A Mendelian randomization analysis of mental and physical health
Source: Medicine (Baltimore). 2025 Nov 7;104(45):e44888. doi: 10.1097/MD.0000000000044888 (PMC12599781; doi:10.1097/MD.0000000000044888)
Supplement: Supplementary file 1 [file medi-104-e44888-s001.docx]

**Supplemental Table 1**. Study details for the genome-wide association studies of exposures and outcomes

| **Type** | **Data** | **Year** | **Characteristic** | **Num of SNP** | **Num of sample** |
| --- | --- | --- | --- | --- | --- |
| exposure | ukb-b-13423 | 2018 | breastfed as a baby | 9851867 | 251150 |
| outcome | ukb-d-ASTHMA_CHILD | 2018 | childhood asthma (age<16) | 10443939 | 361194 |
| outcome | ebi-a-GCST001837 | 2013 | intelligence (childhood) | 1374543 | 12441 |
| outcome | finn-b-KRA_PSY_CHILDSOC | 2021 | social disorders starting during childhood or adolecense | 16380466 | 218792 |
| outcome | ieu-a-1035 | 2013 | difference in height between childhood and adulthood | 2390840 | 5756 |
| outcome | ieu-a-1036 | 2013 | difference in height between childhood and adulthood | 2391721 | 5043 |
| outcome | ieu-a-1037 | 2013 | difference in height between childhood and adulthood | 22384832 | 10799 |
| outcome | ieu-a-1096 | 2012 | childhood obesity | 2442739 | 13848 |
| outcome | ieu-a-16 | 2014 | Childhood intelligence | 1380159 | 12441 |
| outcome | finn-b-CHILDHOOD_ALLERGY | 2021 | childhood allergy (age < 16) | 16380466 | 218792 |
| outcome | finn-b-F5_EMOCHILD | 2021 | emotional disorders and disorders of social functioning with onset specific to childhood | 16380458 | 216650 |
| outcome | ieu-b-13 | 2018 | childhood absence epilepsy | 4979765 | 30470 |
| outcome | ukb-b-13246 | 2018 | childhood sunburn occasions | 9851867 | 346955 |
| outcome | ukb-d-ASTHMA_CHILD | 2018 | childhood asthma (age<16) | 10443939 | 361194 |
| outcome | ukb-e-1737_AFR | 2020 | childhood sunburn occasions | 15537364 | 5809 |
| outcome | ukb-e-1737_CSA | 2020 | childhood sunburn occasions | 199809387 | 7038 |

**Supplemental Table 2**: SNPs of breastfeeding

| SNPs | other_allele | effect_allele | beta | se | pval | mr_keep |
| --- | --- | --- | --- | --- | --- | --- |
| rs3132604 | C | T | -0.00746 | 0.001337 | 2.40E-08 | TRUE |
| rs3095335 | G | C | -0.00746 | 0.001337 | 2.50E-08 | TRUE |
| rs3131921 | T | C | -0.00785 | 0.001439 | 5.00E-08 | TRUE |
| rs2532923 | G | A | -0.0082 | 0.001437 | 1.20E-08 | TRUE |
| rs2844642 | G | A | -0.00771 | 0.001392 | 3.10E-08 | TRUE |
| rs2535296 | A | G | -0.00793 | 0.00138 | 9.10E-09 | TRUE |
| rs9353208 | G | T | -0.00682 | 0.001204 | 1.40E-08 | TRUE |
| rs6919385 | C | T | -0.0068 | 0.001204 | 1.60E-08 | TRUE |
| rs1475430 | G | A | -0.00683 | 0.001204 | 1.40E-08 | TRUE |
| rs9362076 | A | G | -0.00684 | 0.001204 | 1.30E-08 | TRUE |
| rs7759428 | T | C | -0.00685 | 0.001204 | 1.30E-08 | TRUE |
| rs6929228 | T | C | -0.00684 | 0.001204 | 1.30E-08 | TRUE |
| rs9344414 | A | G | -0.00684 | 0.001204 | 1.30E-08 | TRUE |
| rs9362080 | C | A | -0.00684 | 0.001204 | 1.30E-08 | TRUE |
| rs11763279 | C | A | -0.00675 | 0.001238 | 4.90E-08 | TRUE |
| rs10954394 | A | G | -0.00676 | 0.001238 | 4.70E-08 | TRUE |
| rs12539910 | T | C | -0.00675 | 0.001238 | 4.90E-08 | TRUE |
| rs11982852 | C | T | -0.00693 | 0.00125 | 3.00E-08 | TRUE |
| rs6467450 | G | A | -0.0069 | 0.001246 | 3.00E-08 | TRUE |
| rs7806045 | T | C | -0.0068 | 0.001246 | 4.90E-08 | TRUE |
| rs59255824 | C | T | -0.00686 | 0.001247 | 3.80E-08 | TRUE |
| rs62465286 | G | A | -0.00681 | 0.001247 | 4.70E-08 | TRUE |
| rs6962471 | G | A | -0.00686 | 0.00125 | 4.10E-08 | TRUE |
| rs56182580 | T | C | -0.00679 | 0.001195 | 1.30E-08 | TRUE |
| rs1763285 | G | A | -0.00636 | 0.001163 | 4.60E-08 | TRUE |
| rs8010494 | C | T | -0.00649 | 0.001164 | 2.50E-08 | TRUE |
| rs8010613 | C | T | -0.0065 | 0.001164 | 2.40E-08 | TRUE |
| rs1304068 | G | C | -0.0066 | 0.001182 | 2.40E-08 | TRUE |
| rs759978 | G | A | 0.006554 | 0.001136 | 8.10E-09 | TRUE |
| rs28535822 | T | C | 0.006546 | 0.001137 | 8.50E-09 | TRUE |
| rs79954110 | A | C | 0.006663 | 0.001161 | 9.60E-09 | TRUE |
| rs2003618 | A | C | 0.006572 | 0.001137 | 7.50E-09 | TRUE |
| rs9925536 | G | C | 0.006752 | 0.001141 | 3.20E-09 | TRUE |
| rs981783 | G | A | 0.006564 | 0.001202 | 4.70E-08 | TRUE |
| rs4575516 | A | G | 0.006703 | 0.001165 | 8.80E-09 | TRUE |
| rs9928879 | T | G | 0.006577 | 0.001201 | 4.40E-08 | TRUE |
| rs9929064 | T | G | 0.00662 | 0.001202 | 3.60E-08 | TRUE |
| rs9936166 | G | A | 0.006628 | 0.001203 | 3.50E-08 | TRUE |
| rs9929158 | T | C | 0.006616 | 0.001203 | 3.80E-08 | TRUE |
| rs1868975 | A | G | 0.006634 | 0.001202 | 3.40E-08 | TRUE |
| rs1868974 | C | T | 0.00665 | 0.001202 | 3.20E-08 | TRUE |
| rs2717093 | C | T | 0.006151 | 0.001121 | 4.10E-08 | TRUE |
| rs1567820 | C | T | 0.006287 | 0.001121 | 2.00E-08 | TRUE |
| rs1355828 | C | T | 0.006168 | 0.001121 | 3.70E-08 | TRUE |
| rs1015325 | G | A | 0.006138 | 0.001121 | 4.30E-08 | TRUE |
| rs2670567 | G | A | 0.00614 | 0.001121 | 4.30E-08 | TRUE |
| rs2848800 | T | C | 0.006164 | 0.00112 | 3.80E-08 | TRUE |
| rs2120683 | G | C | 0.006133 | 0.001121 | 4.50E-08 | TRUE |
| rs80063801 | C | A | 0.006217 | 0.001121 | 2.90E-08 | TRUE |

**Supplemental Table 3** Details for the SNPs of exposures and outcomes.

| exposure | outcome | | id.exposure | | id.outcome | | | SNP | | p |  |
| --- | --- | --- | --- | --- | --- | --- | --- | --- | --- | --- | --- |
| Breastfeeding | Intelligence | | ukb-b-13423 | | ebi-a-GCST001837 | | | rs1567820 | | 0.139512 |  |
| Breastfeeding | Intelligence | | ukb-b-13423 | | ebi-a-GCST001837 | | | rs8010613 | | 0.970673 |  |
| Breastfeeding | Intelligence | | ukb-b-13423 | | ebi-a-GCST001837 | | | rs9362076 | | 0.327357 |  |
| Breastfeeding | Intelligence | | ukb-b-13423 | | ebi-a-GCST001837 | | | All - Inverse variance weighted | | 0.16507 |  |
| Breastfeeding | Intelligence | | ukb-b-13423 | | ebi-a-GCST001837 | | | All - MR Egger | | 0.893341 |  |
| Breastfeeding | Social disorders | | ukb-b-13423 | | finn-b-KRA_PSY_CHILDSOC | | | rs1567820 | | 0.830785 |  |
| Breastfeeding | Social disorders | | ukb-b-13423 | | finn-b-KRA_PSY_CHILDSOC | | | rs2535296 | | 0.343649 |  |
| Breastfeeding | Social disorders | | ukb-b-13423 | | finn-b-KRA_PSY_CHILDSOC | | | rs56182580 | | 0.364191 |  |
| Breastfeeding | Social disorders | | ukb-b-13423 | | finn-b-KRA_PSY_CHILDSOC | | | rs8010613 | | 0.128459 |  |
| Breastfeeding | Social disorders | | ukb-b-13423 | | finn-b-KRA_PSY_CHILDSOC | | | rs9362076 | | 0.9777 |  |
| Breastfeeding | Social disorders | | ukb-b-13423 | | finn-b-KRA_PSY_CHILDSOC | | | rs9925536 | | 0.028551 |  |
| Breastfeeding | Social disorders | | ukb-b-13423 | | finn-b-KRA_PSY_CHILDSOC | | | All - Inverse variance weighted | | 0.781235 |  |
| Breastfeeding | Social disorders | | ukb-b-13423 | | finn-b-KRA_PSY_CHILDSOC | | | All - MR Egger | | 0.508484 |  |
| Breastfeeding | Height | | ukb-b-13423 | | ieu-a-1035 | | | rs1567820 | | 0.817752 |  |
| Breastfeeding | Height | | ukb-b-13423 | | ieu-a-1035 | | | rs8010613 | | 0.553558 |  |
| Breastfeeding | Height | | ukb-b-13423 | | ieu-a-1035 | | | rs9362076 | | 0.772651 |  |
| Breastfeeding | Height | | ukb-b-13423 | | ieu-a-1035 | | | All - Inverse variance weighted | | 0.62392 |  |
| Breastfeeding | Height | | ukb-b-13423 | | ieu-a-1035 | | | All - MR Egger | | 0.974566 |  |
| Breastfeeding | Height | | ukb-b-13423 | | ieu-a-1036 | | | rs1567820 | | 0.039157 |  |
| Breastfeeding | Height | | ukb-b-13423 | | ieu-a-1036 | | | rs8010613 | | 0.328939 |  |
| Breastfeeding | Height | | ukb-b-13423 | | ieu-a-1036 | | | rs9362076 | | 0.055586 |  |
| Breastfeeding | Height | | ukb-b-13423 | | ieu-a-1036 | | | All - Inverse variance weighted | | 0.882735 |  |
| Breastfeeding | Height | | ukb-b-13423 | | ieu-a-1036 | | | All - MR Egger | | 0.215788 |  |
| Breastfeeding | Height | | ukb-b-13423 | | ieu-a-1037 | | | rs1567820 | | 0.221461 |  |
| Breastfeeding | Height | | ukb-b-13423 | | ieu-a-1037 | | | rs8010613 | | 0.82555 |  |
| Breastfeeding | Height | | ukb-b-13423 | | ieu-a-1037 | | | rs9362076 | | 0.130956 |  |
| Breastfeeding | Height | | ukb-b-13423 | | ieu-a-1037 | | | All - Inverse variance weighted | | 0.912178 |  |
| Breastfeeding | Height | | ukb-b-13423 | | ieu-a-1037 | | | All - MR Egger | | 0.302127 |  |
| Breastfeeding | Obesity | | ukb-b-13423 | | ieu-a-1096 | | | rs1567820 | | 0.933103 |  |
| Breastfeeding | Obesity | | ukb-b-13423 | | ieu-a-1096 | | | rs8010613 | | 0.127531 |  |
| Breastfeeding | Obesity | | ukb-b-13423 | | ieu-a-1096 | | | rs9362076 | | 0.206662 |  |
| Breastfeeding | Obesity | | ukb-b-13423 | | ieu-a-1096 | | | All - Inverse variance weighted | | 0.706731 |  |
| Breastfeeding | Obesity | | ukb-b-13423 | | ieu-a-1096 | | | All - MR Egger | | 0.637586 |  |
| Breastfeeding | intelligence | | ukb-b-13423 | | ieu-a-16 | | | rs1567820 | | 0.139512 |  |
| Breastfeeding | intelligence | | ukb-b-13423 | | ieu-a-16 | | | rs8010613 | | 0.970673 |  |
| Breastfeeding | intelligence | | ukb-b-13423 | | ieu-a-16 | | | rs9362076 | | 0.327357 |  |
| Breastfeeding | intelligence | | ukb-b-13423 | | ieu-a-16 | | | All - Inverse variance weighted | | 0.16507 |  |
| Breastfeeding | intelligence | | ukb-b-13423 | | ieu-a-16 | | | All - MR Egger | | 0.893341 |  |
| Breastfeeding | | allergy (age < 16) | | ukb-b-13423 | | finn-b-CHILDHOOD_ALLERGY | rs1567820 | | 0.653954 | | |
| Breastfeeding | | allergy (age < 16) | | ukb-b-13423 | | finn-b-CHILDHOOD_ALLERGY | rs2535296 | | 0.242032 | | |
| Breastfeeding | | allergy (age < 16) | | ukb-b-13423 | | finn-b-CHILDHOOD_ALLERGY | rs56182580 | | 0.451875 | | |
| Breastfeeding | | allergy (age < 16) | | ukb-b-13423 | | finn-b-CHILDHOOD_ALLERGY | rs8010613 | | 0.217788 | | |
| Breastfeeding | | allergy (age < 16) | | ukb-b-13423 | | finn-b-CHILDHOOD_ALLERGY | rs9362076 | | 0.321562 | | |
| Breastfeeding | | allergy (age < 16) | | ukb-b-13423 | | finn-b-CHILDHOOD_ALLERGY | rs9925536 | | 0.66269 | | |
| Breastfeeding | | allergy (age < 16) | | ukb-b-13423 | | finn-b-CHILDHOOD_ALLERGY | All - Inverse variance weighted | | 0.587504 | | |
| Breastfeeding | | allergy (age < 16) | | ukb-b-13423 | | finn-b-CHILDHOOD_ALLERGY | All - MR Egger | | 0.234754 | | |
| Breastfeeding | | Emotional disorders | | ukb-b-13423 | | finn-b-F5_EMOCHILD | rs1567820 | | 0.783207 | | |
| Breastfeeding | | Emotional disorders | | ukb-b-13423 | | finn-b-F5_EMOCHILD | rs2535296 | | 0.57782 | | |
| Breastfeeding | | Emotional disorders | | ukb-b-13423 | | finn-b-F5_EMOCHILD | rs56182580 | | 0.316412 | | |
| Breastfeeding | | Emotional disorders | | ukb-b-13423 | | finn-b-F5_EMOCHILD | rs8010613 | | 0.606062 | | |
| Breastfeeding | | Emotional disorders | | ukb-b-13423 | | finn-b-F5_EMOCHILD | rs9362076 | | 0.997186 | | |
| Breastfeeding | | Emotional disorders | | ukb-b-13423 | | finn-b-F5_EMOCHILD | rs9925536 | | 0.619601 | | |
| Breastfeeding | | Emotional disorders | | ukb-b-13423 | | finn-b-F5_EMOCHILD | All - Inverse variance weighted | | 0.597984 | | |
| Breastfeeding | | Emotional disorders | | ukb-b-13423 | | finn-b-F5_EMOCHILD | All - MR Egger | | 0.471111 | | |
| Breastfeeding | | absence epilepsy | | ukb-b-13423 | | ieu-b-13 | rs1567820 | | 0.04419 | | |
| Breastfeeding | | absence epilepsy | | ukb-b-13423 | | ieu-b-13 | rs56182580 | | 0.928337 | | |
| Breastfeeding | | absence epilepsy | | ukb-b-13423 | | ieu-b-13 | rs8010613 | | 0.00067 | | |
| Breastfeeding | | absence epilepsy | | ukb-b-13423 | | ieu-b-13 | rs9362076 | | 0.969051 | | |
| Breastfeeding | | absence epilepsy | | ukb-b-13423 | | ieu-b-13 | rs9925536 | | 0.077177 | | |
| Breastfeeding | | absence epilepsy | | ukb-b-13423 | | ieu-b-13 | All - Inverse variance weighted | | 0.893968 | | |
| Breastfeeding | | absence epilepsy | | ukb-b-13423 | | ieu-b-13 | All - MR Egger | | 0.97848 | | |
| Breastfeeding | | sunburn | | ukb-b-13423 | | ukb-b-13246 | rs1567820 | | 0.001885 | | |
| Breastfeeding | | sunburn | | ukb-b-13423 | | ukb-b-13246 | rs2535296 | | 1.74E-05 | | |
| Breastfeeding | | sunburn | | ukb-b-13423 | | ukb-b-13246 | rs56182580 | | 0.799514 | | |
| Breastfeeding | | sunburn | | ukb-b-13423 | | ukb-b-13246 | rs8010613 | | 0.331522 | | |
| Breastfeeding | | sunburn | | ukb-b-13423 | | ukb-b-13246 | rs9362076 | | 0.539337 | | |
| Breastfeeding | | sunburn | | ukb-b-13423 | | ukb-b-13246 | rs9925536 | | 0.522097 | | |
| Breastfeeding | | sunburn | | ukb-b-13423 | | ukb-b-13246 | All - Inverse variance weighted | | 0.094158 | | |
| Breastfeeding | | sunburn | | ukb-b-13423 | | ukb-b-13246 | All - MR Egger | | 0.367611 | | |
| Breastfeeding | | asthma (age<16) | | ukb-b-13423 | | ukb-d-ASTHMA_CHILD | rs1567820 | | 0.745366 | | |
| Breastfeeding | | asthma (age<16) | | ukb-b-13423 | | ukb-d-ASTHMA_CHILD | rs2535296 | | 0.000479 | | |
| Breastfeeding | | asthma (age<16) | | ukb-b-13423 | | ukb-d-ASTHMA_CHILD | rs56182580 | | 0.110963 | | |
| Breastfeeding | | asthma (age<16) | | ukb-b-13423 | | ukb-d-ASTHMA_CHILD | rs8010613 | | 0.32586 | | |
| Breastfeeding | | asthma (age<16) | | ukb-b-13423 | | ukb-d-ASTHMA_CHILD | rs9362076 | | 0.095229 | | |
| Breastfeeding | | asthma (age<16) | | ukb-b-13423 | | ukb-d-ASTHMA_CHILD | rs9925536 | | 0.901749 | | |
| Breastfeeding | | asthma (age<16) | | ukb-b-13423 | | ukb-d-ASTHMA_CHILD | All - Inverse variance weighted | | 0.033134 | | |
| Breastfeeding | | asthma (age<16) | | ukb-b-13423 | | ukb-d-ASTHMA_CHILD | All - MR Egger | | 0.042072 | | |
| Breastfeeding | | sunburn | | ukb-b-13423 | | ukb-e-1737_AFR | rs1567820 | | 0.140592 | | |
| Breastfeeding | | sunburn | | ukb-b-13423 | | ukb-e-1737_AFR | rs2535296 | | 0.835911 | | |
| Breastfeeding | | sunburn | | ukb-b-13423 | | ukb-e-1737_AFR | rs56182580 | | 0.932235 | | |
| Breastfeeding | | sunburn | | ukb-b-13423 | | ukb-e-1737_AFR | rs8010613 | | 0.266627 | | |
| Breastfeeding | | sunburn | | ukb-b-13423 | | ukb-e-1737_AFR | rs9362076 | | 0.427115 | | |
| Breastfeeding | | sunburn | | ukb-b-13423 | | ukb-e-1737_AFR | rs9925536 | | 0.956078 | | |
| Breastfeeding | | sunburn | | ukb-b-13423 | | ukb-e-1737_AFR | All - Inverse variance weighted | | 0.642049 | | |
| Breastfeeding | | sunburn | | ukb-b-13423 | | ukb-e-1737_AFR | All - MR Egger | | 0.338858 | | |
| Breastfeeding | | sunburn | | ukb-b-13423 | | ukb-e-1737_CSA | rs1567820 | | 0.962321 | | |
| Breastfeeding | | sunburn | | ukb-b-13423 | | ukb-e-1737_CSA | rs2535296 | | 0.254993 | | |
| Breastfeeding | | sunburn | | ukb-b-13423 | | ukb-e-1737_CSA | rs56182580 | | 0.391128 | | |
| Breastfeeding | | sunburn | | ukb-b-13423 | | ukb-e-1737_CSA | rs8010613 | | 0.963983 | | |
| Breastfeeding | | sunburn | | ukb-b-13423 | | ukb-e-1737_CSA | rs9362076 | | 0.815033 | | |
| Breastfeeding | | sunburn | | ukb-b-13423 | | ukb-e-1737_CSA | rs9925536 | | 0.284496 | | |
| Breastfeeding | | sunburn | | ukb-b-13423 | | ukb-e-1737_CSA | All - Inverse variance weighted | | 0.691583 | | |
| Breastfeeding | | sunburn | | ukb-b-13423 | | ukb-e-1737_CSA | All - MR Egger | | 0.433723 | | |

**Supplemental Table 4** Heterogeneity of IVW and MR Egge between breastfeed and childhood asthma

| **exposure** | **outcome** | **method** | **Q** | **Q_pval** |
| --- | --- | --- | --- | --- |
| ukb-b-13423 | ukb-d-ASTHMA_CHILD | MR Egger | 2.213 | 0.697 |
| ukb-b-13423 | ukb-d-ASTHMA_CHILD | IVW | 9.754 | 0.0825 |

**Supplemental Table 5** Horizontal pleiotropy analysis of MR Egger between breastfeed and childhood asthma

| **exposure** | **outcome** | **egger_intercept** | **pval** |
| --- | --- | --- | --- |
| ukb-b-13423 | ukb-d-ASTHMA_CHILD | 0.003 | 0.0516 |
